# Biochemical Studies of the Lagunamides, Potent Cytotoxic Cyclic Depsipeptides from the Marine Cyanobacterium *Lyngbya majuscula*

**DOI:** 10.3390/md10051126

**Published:** 2012-05-23

**Authors:** Ashootosh Tripathi, Wanru Fang, David Tai Leong, Lik Tong Tan

**Affiliations:** 1 Natural Sciences and Science Education, National Institute of Education, Nanyang Technological University, 1 Nanyang Walk, 637616, Singapore; Email: ashootosh.tripathi@gmail.com; 2 Department of Chemical and Biomolecular Engineering, National University of Singapore, 4 Engineering Drive, 117576, Singapore; Email: chefangw@nus.edu.sg

**Keywords:** marine cyanobacterium, cyclic depsipeptides, lagunamides, apoptosis, cytotoxicity

## Abstract

Lagunamides A (**1**) and B (**2**) are potent cytotoxic cyclic depsipeptides isolated from the filamentous marine cyanobacterium, *Lyngbya majuscula*, from Pulau Hantu, Singapore. These compounds are structurally related to the aurilide-class of molecules, which have been reported to possess exquisite antiproliferative activities against cancer cells. The present study presents preliminary findings on the selectivity of lagunamides against various cancer cell lines as well as their mechanism of action by studying their effects on programmed cell death or apoptosis. Lagunamide A exhibited a selective growth inhibitory activity against a panel of cancer cell lines, including P388, A549, PC3, HCT8, and SK-OV3 cells, with IC_50_ values ranging from 1.6 nM to 6.4 nM. Morphological studies showed blebbing at the surface of cancer cells as well as cell shrinkage accompanied by loss of contact with the substratum and neighboring cells. Biochemical studies using HCT8 and MCF7 cancer cells suggested that the cytotoxic effect of **1** and **2** might act via induction of mitochondrial mediated apoptosis. Data presented in this study warrants further investigation on the mode of action and underscores the importance of the lagunamides as potential anticancer agents.

## 1. Introduction

Benthic filamentous marine cyanobacteria are a source of novel bioactive compounds. To date more than 400 nitrogen-containing molecules have been reported in the literature, belonging mostly to the polyketide-polypeptide structural class of natural products [1]. The importance of this structural class is attested by a number of natural products, including bleomycin, rapamycin, and FK506, currently being used as pharmaceuticals [2]. A high proportion of marine cyanobacterial compounds, such as dolastatin 10, apratoxin A, and largazole, have shown to possess exquisite potency and have subsequently been considered for further development as therapeutic agents, especially in the area of cancer research. The high potencies of these compounds are due to their specific interference with cellular targets, including microtubules, actin filaments, and histone deacetylase [3]. To date, a dolastatin 10-analogue, auristatin E, has been formulated as an antibody drug conjugate, brentuximab vedotin, and approved for the treatment of Hodgkin lymphoma and anaplastic large cell lymphoma [4]. In addition, at least three antibody drug conjugates based on synthetic analogues of marine cyanobacterial compounds are currently in the clinical pipeline as anticancer agents [5].

Recent research by Tan and co-workers revealed the chemical richness of a persistent strain of the marine cyanobacterium, *Lyngbya majuscula*, collected from Pulau Hantu, Singapore. At least a dozen compounds, both new (e.g. hantupeptins and besarhanamides) and known have been reported from this strain [6,7,8]. A recollection of this particular cyanobacterial strain in 2007 yielded two cyclodepsipeptides, lagunamides A and B, with significant cytotoxicity having IC_50_ values of 6.4 and 20.5 nM, respectively, when tested against the P388 murine leukemia cell line [9]. These compounds belong to the polyketide-polypeptide natural products and are structurally related to the aurilide-class of molecules. In addition, significant antimalarial activities were also reported for the lagunamides, a first for this class of molecules [9]. Due to the exquisite potency of the lagunamides as well as being available in sufficient quantities, they were selected for further biological testing, in particular, for their effects on apoptosis or cell death.

The apoptotic pathway has become a major target for developing anticancer drugs due to the conserved pathway of programmed cell death [10]. During embryonic development, cell death is essential for successful organogenesis and the formation of complex multicellular tissues [11]. Apoptosis also operates in adult organisms to maintain normal cellular homeostasis, and resistance to apoptosis is a hallmark of cancer, often contributing to chemoresistance. Several key apoptotic pathways are altered in cancer, including the “loss of function” mutations in the p53 tumor suppressor gene, as well as the overexpression of anti-apoptotic proteins such as the mitochondrial anti-oncogenes Bcl-2 and Bcl-X_L_ [12]. Herein are preliminary biological data conducted on the lagunamides to profile their cytotoxic activities against a panel of cancer cell lines as well as their effect on the apoptotic pathway based on a biochemical method.

## 2. Results and Discussion

### 2.1. Structures of Lagunamides and the Aurilide-Class of Molecules

**Chart 1:**
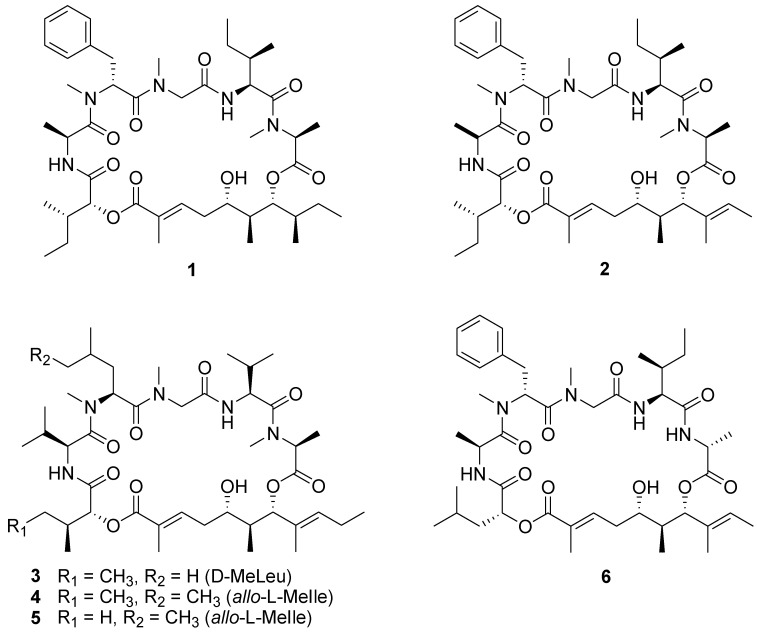
Chemical structures of lagunamides (**1** and **2**), aurilides (**3**–**5**), and kulokekahilide-2 (**6**).

Lagunamides A (**1**) and B (**2**) (Chart 1) are cyclic depsipeptides recently isolated from the marine cyanobacterium, *Lyngbya majuscula*, obtained from the shallow lagoon at Pulau Hantu, Singapore [9]. The planar structures were established primarily by 1D and 2D NMR data as well as MS spectral data. These compounds belong to the mixed polyketide-polypeptide structural class, consisting of five amino acids (Ala, *N*Me-Phe, *N*Me-Gly, Ile, and *N*Me-Ala), one-hydroxy acid (2-hydroxy-isoleucic acid) and a unique extended polyketide moiety. The lagunamides are structurally related to the aurilide class of molecules, which include aurilides B (**4**) and C (**5**) and kulokekahilide-2 (**6**) (Chart 1) [13,14,15]. Aurilide (**3**), the first member of this class of molecules, was originally reported from the sea hare, *Dolabella auricularia* [16]. However, it has been speculated that marine cyanobacteria are the true source of the compound and the presence of aurilide in the animal is due to sequestration via the invertebrate’s diet. There is growing interest in the development of these molecules as potential anticancer agents as attested by a number of synthetic efforts geared towards their total synthesis as well as generation of analogues for structural activity relationship (SAR) studies [15,17,18,19,20,21].

### 2.2. Evaluation of Lagunamide A against BJ and BJ shp53 Cell Lines

**Figure 1:**
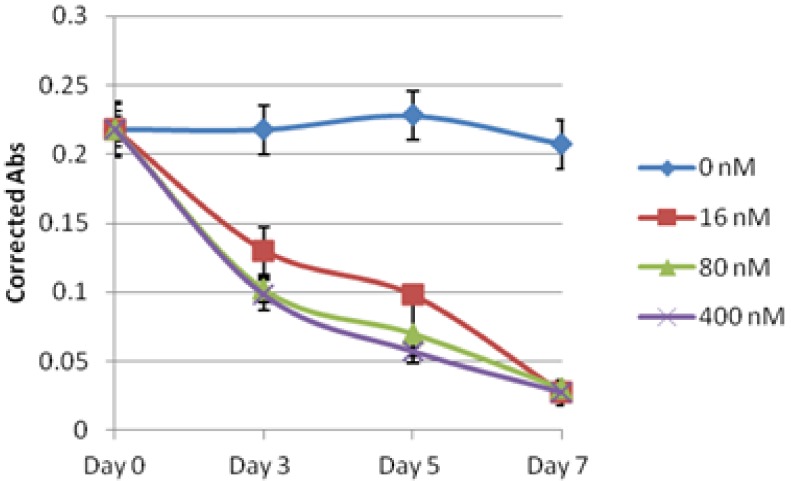
Cytotoxic effects of lagunamide A (**1**) against BJ cells over 7 days.

**Figure 2:**
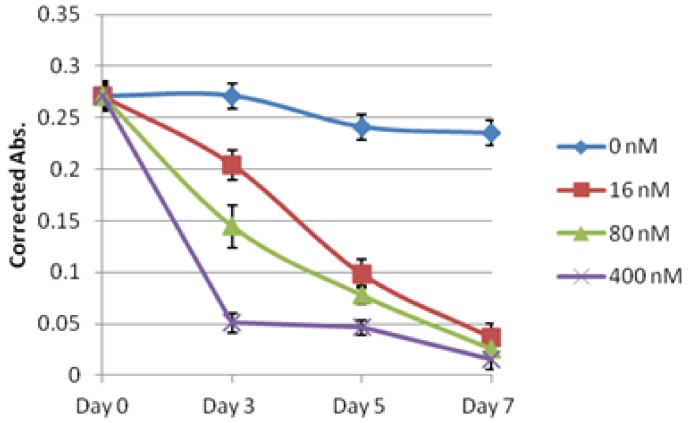
Cytotoxic effects of lagunamide A (**1**) against BJ shp53 cells over 7 days.

Lagunamide A (**1**) was originally reported to induce cytotoxicity in the P388 murine leukemia cell line with an IC_50_ of 6.4 nM [9]. Subsequently, it was decided to first test **1** against human foreskin fibroblast cells (BJ) and p53 tumor suppressor oncogene knocked down fibroblast cells (BJ shp53). The rationale was to elucidate if the cytotoxic nature of **1 **was specific towards normal cell types and also to determine if p53 expression could account for any alteration in activities. Compound **1 **displayed IC_50_ values of 20.2 and 58.8 nM when tested against BJ and BJ shp53 cells, respectively (Table 1). The differential IC_50 _values observed in the two cell lines suggested that the p53 gene may play a role in the sensitivity of BJ cells to **1**, as disabling p53 function in BJ shp53 reduced the sensitivity by almost 3-fold. Furthermore, time-based experiments carried out on both cell types showed that BJ shp53 (Figure 1 and Figure 2) responded more sensitively when treated at 400 nM over a short span of time of 3 days. However, at other concentrations, **1** caused a significant reduction in proliferation of both BJ and BJ shp53 cells. 

**Table 1:** IC_50 _values (nM) of lagunamides A (**1**) and B (**2**), aurilide (**3**), aurilides B (**4**) and C (**5**), and kulokekahilide-2 (**6**) against various cell lines.

Cell Lines	Cell Types	1	2	3 *^a^*	4 *^b^*	5 *^b^*	6 *^c^*
BJ	Foreskin fibroblast	20.2					
BJ shp53	p53 knocked down fibroblast	58.8					
P388	Murine leukemia	6.4	20.5				4.2
A549	Lung adenocarcinoma epithelial	2.9					
NCI-H460	Lung large-cell carcinoma				10	50	
PC3	Prostate carcinoma	2.5					
HCT8	Ileocecal colorectal adenocarcinoma	1.6	5.2				
SK-OV3	Ovarian carcinoma	3.8					7.5
HeLaS3	Human epithelial carcinoma			11			
MDA-MB-435	Human breast carcinoma						14.6
A10	Vascular smooth muscle						59.1
Neuro-2a	Mouse neuroblastoma				40	130	

*^a^* Cytotoxic data obtained from Suenaga *et al.* [17]; *^b^* cytotoxic data obtained from Han *et al.* [14]; *^c^* cytotoxic data obtained from Nakao *et al*. [13].

The p53 suppresser oncogene is known to guard against DNA damage, intracellular stresses, and to modulate proliferative signals, thus preventing cells from turning malignant by inducing either cell arrest or apoptosis [22]. Furthermore, there are several pro-apoptotic transcriptional targets of p53, such as BAX and BH-3 proteins, and NOXA and PUMA, which promote cytochrome *c* release from the mitochondrion [23]. The activity of **1** on fibroblast cells indicates that it might act by stressing the mitochondrion through p53 up-regulation leading to the initiation of the intrinsic apoptotic pathway.

There are generally two pathways that trigger apoptosis: the extrinsic and intrinsic apoptotic pathways [24]. The extrinsic pathway, otherwise known as the “death receptor pathway”, is characterized by the activation of death receptors on the cell surface, leading to the initiation of the caspase cascade via caspase 8. In contrast, the intrinsic pathway involves the permeabilization of mitochondria and release of cytochrome *c* from mitochondria into the cytoplasm, leading to the activation of the caspase cascade through caspase 9. Whichever pathway is taken, both lead to the activation of various caspase-enzymes responsible for the demise of the cell. The majority of apoptosis in vertebrate systems proceeds through the mitochondrial pathway [25].

### 2.3. Evaluation of Lagunamide A against a Panel of Cancer Cell Lines

Lagunamide A (**1**) was next tested against a panel of cancer cell lines, including P388, A549, PC3, HCT8, and SK-OV3 (Table 1). The cytotoxicity data indicated that these cancer cells were more susceptible to **1** by approximately four to ten-fold compared to the BJ (Table 1). Due to limited amounts of lagunamide B (**2**), it was tested only in two cell lines, P388 and HCT8, which showed IC_50_ values of 20.5 and 5.2 nM, respectively. Comparing the two lagunamides, **2** showed reduced cytotoxicity compared to **1**. Since the only structural difference between **1** and **2** is presence of the olefinic group in the latter molecule, the enhanced cytotoxicity of compound **1** could be due to the non specific toxicity arising from the conjugate addition of cellular nucleophiles by **2** [26]. In addition, the human A549 lung adenocarcinoma epithelial cells used in the assay were reported not to undergo Fas-mediated apoptosis [27]. However, it was observed in this study that A549 cells were sensitive to the cytotoxic effect of **1**, with an IC_50_ value of 2.9 nM, suggesting that **1** might not induce apoptosis through the extrinsic pathway for killing cancer cells.

Selective cytotoxicity towards other cell lines was also observed for other members of the aurilide class of molecules (Table 1). For instance, the IC_50_ values of kulokekahilide-2 against several cell lines, including P388, SK-OV-3, MDA-MB-435, and A-10, ranged from 4.2 to 59.1 nM [13]. When evaluated in the NCI 60 cell lines, aurilide and aurilide B were selective against renal, and prostate cancer cell lines [14,17]. Furthermore, both aurilide and aurilides B and C showed significant *in vivo* antitumor activities based on NCI’s hollow fiber assay [14,17]. Given the profile of potent cytotoxicity to cancer cells, the lagunamides join the growing list of aurilide-class molecules as potential leads in cancer treatment. 

**Figure 3:**
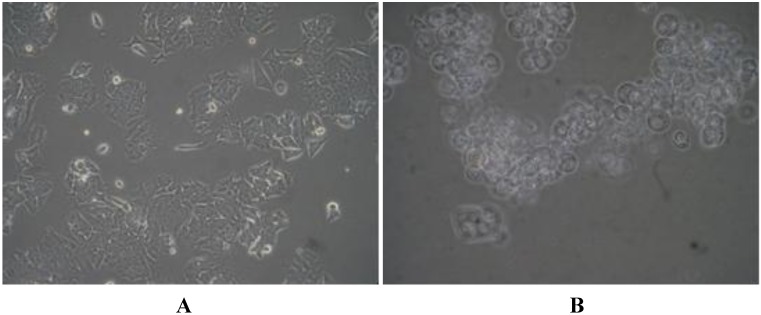
Microscopic (×200) images of (**A**) untreated HCT 8 cells and (**B**) HCT 8 cells treated with lagunamide A (**1**).

Morphological observations revealed that the loss of cell proliferation in all cancer cells was associated with cell shrinkage, accompanied by the loss of contact with the substratum and neighboring cells. The detached cells appeared to be spherical with blebbing morphology when observed under high magnifications (Figure 3). Such morphological observations are the hallmark indicators of programmed cell death/apoptosis [28].

### 2.4. Biochemical Studies of Lagunamide A Using HCT8 and MCF7 Cell Lines

**Figure 4:**
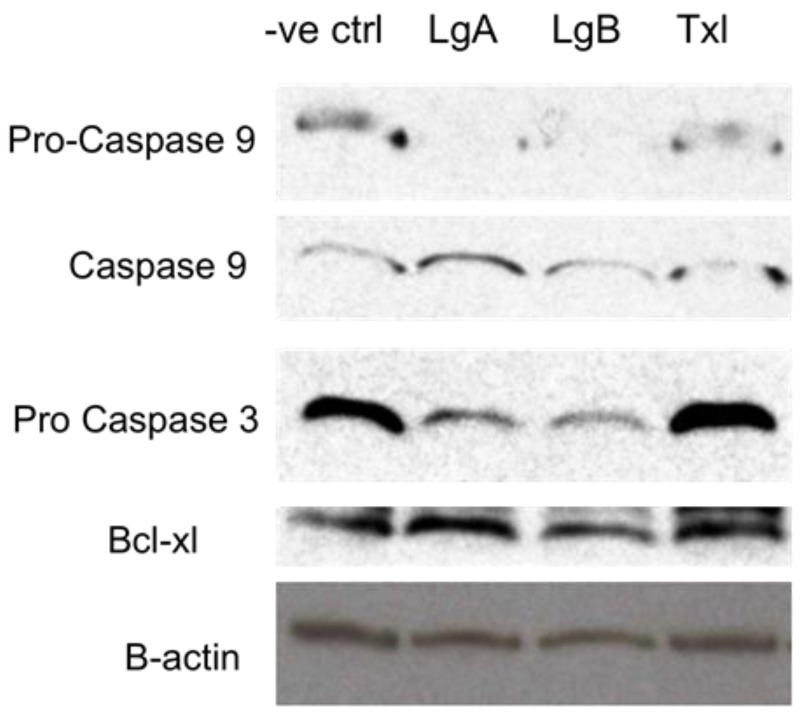
Western blot analysis of lysates from DMSO (-ve ctrl), lagunamide A (Lg A), lagunamide B (Lg B), and taxol (Txl)-treated HCT8 cells with pro-caspase 9, caspase 9, pro-caspase and Bcl-X_L_ antibodies. The anti-actin antibody was used to ensure equal amounts of protein were loaded.

Results thus far were suggestive of the intrinsic apoptotic pathway as the probable mode of action associated with lagunamide A (**1**). An in-depth biochemical analysis was therefore initiated to determine if lagunamides A and B trigger a caspase-mediated apoptosis. As such, Western blot analyses of **1**- or **2**-treated HCT 8 cell lysates with caspase 9, caspase 3 and Bcl-X_L_ antibodies were performed (Figure 4). These experiments revealed that caspase 9 was cleaved and activated, suggesting activation of mitochondrial-mediated apoptosis through the release of cytochrome *c*. In addition, expression level of Bcl-X_L_ (anti-apoptosis) remains fairly constant, suggesting no upregulation of the anti-apoptotic response. **1**-treated HCT 8 cells demonstrated the highest level of cleaved (active fragment) caspase 9 (Figure 4), indicating the involvement of cytochrome *c* as well as the nucleotide dATP/ATP which auto-catalytically activates procaspase 9, as shown by the cleaved caspase 9 band (Figure 4). This, in turn, can proceed to activate downstream executioner caspases, such as caspases-3 and -7.

Subsequently, the level of pro-caspase 3 was observed to be lower for cells exposed to **1** and **2** when compared to both positive (taxol-treated) and negative control (DMSO-treated) cell lysates. This suggested the plausible downstream processing of the apoptotic cascade. Cleaved caspase 3, however, was not detected in the experiment. Interestingly, cells treated with **1** also expressed marginally higher levels of Bcl-X_L_ (a pro-survival factor) compared to **2**.

**Figure 5:**
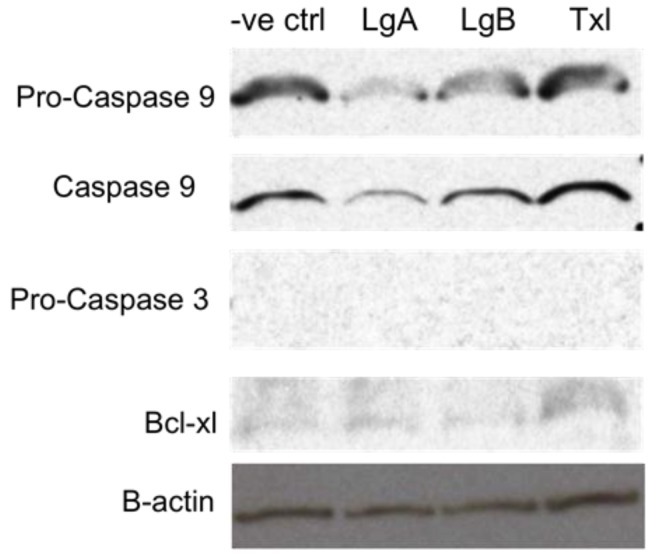
Western blot analysis of lysates from DMSO (-ve ctrl), lagunamide A (Lg A), lagunamide B (Lg B), and taxol (Txl)-treated MCF7 cells with pro-caspase 9, caspase 9, pro-caspase 3 and Bcl-X_L_ antibodies. The anti-actin antibody was used to ensure equal amounts of protein were loaded.

The biochemical study was also extended to MCF7 breast cancer cells treated with **1** and **2** (Figure 5). These experiments revealed that **1**-treated MCF7 cells had the lowest level of caspase 9 compared to cancer cells treated with taxol and DMSO (negative control). The endogenous levels of pro-caspase 3 were undetected in MCF7 cells, suggesting that it had undergone autocatalysis to initiate a further downstream cascade leading to the intrinsic apoptotic pathway. Moreover, unlike in treated HCT8 cells, Bcl-X_L_ was shown to be present at very low levels for all treatments (Figure 5).

These series of biochemical studies suggested that the cytotoxicity of the lagunamides could be due to either passive diffusion or active transported into the cells, triggering mitochondrial-mediated apoptosis. Recently, a report by Sato and co-workers showed that aurilide induces apoptosis via the mitochondrial-mediated pathway [29]. In addition, the study showed aurilide to selectively target prohibitin 1 (PHB 1) localized within the mitochondria. One of the functions of prohibitin is in the maintenance of mitochondrial function as well as protection from senescence [30]. The inhibition of PHB 1 by aurilide led to the induction of the mitochondrial fragmentation through enhanced processing of the mitochondrial fusion protein optic atrophy 1 (OPA1). This resulted in the loss of membrane potential and the induction of apoptotic cell death [29]. Interestingly, they did not report any downregulation/upregulation of caspases in their study. Aurilide represents the first natural product that inhibits the function of prohibitin in the regulation of OPA1 and mitochondria-induced apoptosis.

In summary, further biological testing, including biochemical studies, allowed preliminary investigation of the lagunamides in terms of their cytotoxic activities and effects on programmed cell death. Lagunamide A exhibited selectivity in activity when tested against a panel of cell lines, including P388, A549, PC3, HCT8, and SK-OV3, with IC_50_ values ranging from 1.6 nM to 6.4 nM. Although compound **2** was tested in only two cell lines, it showed lower potency when compared to **1**, indicating the importance of the saturated form in the polyketide moiety of **1**. Biochemical studies using HCT8 and MCF7 cancer cells suggested that the cytotoxic effect of the lagunamides might act via induction of mitochondrial mediated apoptosis. Data presented in this study underscores the importance of the aurilide class of molecules as potential anti-cancer therapeutic agents and also encourages more in-depth investigation into other therapeutic uses of this interesting class of marine derived compounds.

## 3. Experimental Section

### 3.1. Extraction and Isolation of Lagunamides A and B

The filamentous benthic marine cyanobacterium, *Lyngbya majuscula*, was collected from the western lagoon of Pulau Hantu Besar, Singapore on 25 June, 2007. Voucher specimen of this strain is maintained at the National Institute of Education under the code TLT/PHB/002. The isolation and purification of the lagunamides follow that of Tripathi *et al.* [9]. Briefly, samples of the marine cyanobacterium, *Lyngbya majuscula*, stored previously in 70% aqueous EtOH, were extracted exhaustively with 1:1 CHCl_3_:MeOH to yield about 1.0 g of organic extract. The extract was subjected to normal phase Si gel vacuum flash chromatography and the bioactive fractions were identified using the brine shrimp toxicity assay. Upon clean up with RP-18 SEP-PAK, the active sub-fraction was subjected to further purification using RP-18 (Phenomenex Sphereclone 5 μm ODS, 250 × 10.00 mm) Preparative HPLC, eluted with 78:22 MeOH:H_2_O, to provide pure lagunamides A (1) and B (2).

### 3.2. Cancer Cell Lines and Media

HCT8, MCF7, A549, PC3, SK-OV3, P388, and BJ cells were obtained from the American Type Culture Collection (ATCC). BJ shp53 cells were made in-house [31]. Cells and cell lines were maintained in either Dulbecco’s modified Eagle’s medium (DMEM, Gibco, Carlsbad, CA, USA) or RPMI-1640 media, supplemented with 10% fetal bovine serum (FBS, Hyclone, Waltham, MA, USA), 5% L-glutamine (PAA, Pasching, Austria) and 1% penicillin/streptomycin (PAA, Pasching, Austria), incubated at 37 °C and 5% CO_2_.

### 3.3. MTT Cytotoxicity Assay

The cytotoxicity on cells was determined by MTT staining assay, according to an established reported procedure [12,14]. All compounds were dissolved in 100% DMSO at 1 mM stock concentration. In the MTT assay, the compound stock solutions were diluted in cell culture medium containing 10% DMSO and pipetted into cell culture wells. The final DMSO concentration was 1.25% in each well. A standard plating format was used on each microtiter plate. Six serial dilutions were made for each compound in order to generate a dose response curve. Each plate contained a blank well (cell-free medium-only well), solvent control wells which contained cells and 1.25% DMSO, drug control wells which contained drug and medium only and growth control wells which contained only cells in medium. There were three replicate wells for each compound dilution.

Adherent cells were seeded at 20,000 cells per well and cultured for 24 h before exposure to compounds for 24 h. After incubation, cell viability was determined by the MTT dye reduction method [12] and measured at 570 nm using a microtiter plate reader (Biorad, 168-1000EDU). The inhibitory concentration, IC_50_, was defined as the effective concentration of compounds that caused 50% reduction of cell growth relative to solvent controls. Each IC_50_ value was calculated based on the dose response curve by non-linear regression and sigmoidal analysis function in the GraphPad Prism data analysis software (version 5, GraphPad software: La Jolla, CA, USA, 2011).

### 3.4. Western Blot Analysis

Approximately 200,000 HCT8 and MCF7 cells were incubated with 100 nM lagunamides A (**1**), B (**2**), and taxol for 10 h. The cell pellets were collected by centrifugation at 200 g for 5 min at 4 °C, washed three times with PBS and lysed by resuspension in lysis solution (7 M urea, 2 M thiourea, 4% CHAPS, 30 mM Tris, pH 9.0) and brief sonication on ice for 2 min at 30% amplitude, 30 s interval. The cell lysates were centrifuged at 12,000 rpm for 5 min. The protein-containing supernatants from the control untreated cells and treated cell lysates were quantified using Bradford protein assay reagent (Biorad, 500-0201). Equal amounts of proteins (20 mg) were boiled at 95 °C with 10 µL of 2× laemmli sample buffer (Biorad, 161-0737) for 5 min and loaded into each lane of a SDS-PAGE gel. Cell lysates were separated by pre-cast 4–15% SDS-PAGE under reducing conditions and transferred to nitrocellulose membranes. Membranes were blocked with 5% non-fat milk diluted in TBS-T for 1 h at room temperature. The membranes were then probed with rabbit anti-caspase-3 (1:1000), rabbit anti-caspase-9 (1:1000) or rabbit anti-Bcl-xl (1:1000) antibodies (Cell Signaling Technology) at 4 °C overnight. Mouse anti-Beta-actin (1:20,000) (Santa Cruz) was used as a loading control. Incubation with anti-Beta-actin antibodies was for 1 h at room temperature. After washing with TBS-T, the membranes were incubated with goat anti-rabbit HRP-conjugated antibody (1:40,000) (Santa Cruz) or goat anti-mouse HRP conjugated antibody (1:40,000) (Santa Cruz). Specific band signals were detected by chemiluminescence on film.

## 4. Conclusions

Biological studies of the lagunamides, in particular compound **1**, showed selective cytotoxicity against a panel of cancer cell lines, with HCT8 being the most sensitive cell line with an IC_50_ of 1.8 nM. Preliminary data from a series of biochemical experiments using HCT8 and MCF7 cell lines suggested that the cytotoxic effects of the lagunamides operate via the intrinsic apoptotic pathway.
